# Supplementary material for: Correlation between Adverse Events and Antibody Titers among Healthcare Workers Vaccinated with BNT162b2 mRNA COVID-19 Vaccine
Source: Vaccines (Basel). 2022 Jul 30;10(8):1220. doi: 10.3390/vaccines10081220 (PMC9412348; doi:10.3390/vaccines10081220)
Supplement: Supplementary file 1 [file vaccines-10-01220-s001.zip › vaccines-1802640-supplementary.pdf]

Table S1: Adverse events following first and second vaccine doses by age.

	After 1st Vaccination (N=233)			After 2nd Vaccination (N=733)		
	< 55 years old (N=176)	>55 years old (N=57)	P	< 55 years old (N=558)	>55 years old (N=175)	P
At least one adverse event						
Pain at injection site	158 (89.8)	37 (64.9)	<.0001	490 (87.8)	121 (69.1)	<.0001
Heat at injection site	18 (10.2)	2 (3.5)	0.11	50 (9)	12 (6.9)	0.38
Local erythema	13 (7.4)	1 (1.8)	0.12	41 (7.3)	9 (5.1)	0.31
Axillary Lymphadenopathy	2 (1.1)	0 (0)	0.42	40 (7.2)	8 (4.6)	0.22
Fatigue	42 (23.9)	12 (21.1)	0.66	289 (51.8)	68 (38.9)	0.0028
Headache	39 (22.2)	5 (8.8)	0.02	237 (42.5)	41 (23.4)	<.0001
Myalgia	23 (13.1)	7 (12.3)	0.87	229 (41)	43 (24.6)	<.0001
Fever >38°c	2 (1.1)	0 (0)	0.41	45 (8.1)	8 (4.6)	0.12
Arthralgia	3 (1.7)	3 (5.3)	0.14	88 (15.8)	17 (9.7)	0.04
Systemic rash	1 (0.6)	0 (0)	0.56	2 (0.4)	0 (0)	0.42
Pruritus	2 (1.1)	0 (0.0)	0.41	15 (2.7)	2 (1.1)	0.23
Facial paresthesia	1 (0.6)	1 (1.8)	0.39	7 (1.3)	2 (1.1)	0.90
Non facial paresthesia	2 (1.1)	1 (1.8)	0.72	19 (3.4)	4 (2.3)	0.45
Need for antipyretic or analgetic medication				184 (33.0)	37 (21.1)	0.0029

Table S2: Adverse events following first and second vaccine by gender.

	After 1st Vaccination (N=233)			After 2nd Vaccination (N=733)		
	Female (N=179)	Male (N=54)	P	Female (N=557)	Male (N=176)	P
At least one adverse event						
Pain at injection site	156 (87.2)	39 (72.2)	0.0093	483 (86.7)	128 (72.7)	<.0001
Heat at injection site	18 (10.1)	2 (3.7)	0.1441	58 (10.4)	4 (2.3)	0.0007
Local erythema	12 (6.7)	2 (3.7)	0.4161	47 (8.4)	3 (1.7)	0.0020
Axillary Lymphadenopathy	2 (1.1)	0 (0)	0.4353	46 (8.3)	2 (1.1)	0.0009
Fatigue	49 (27.4)	5 (9.3)	0.0057	295 (53)	62 (35.2)	<.0001
Headache	40 (22.3)	4 (7.4)	0.0140	243 (43.6)	35 (19.9)	<.0001
Myalgia	25 (14)	5 (9.3)	0.3653	238 (42.7)	34 (19.3)	<.0001
Fever >38°c	2 (1.1)	0 (0)	0.4353	44 (7.9)	9 (5.1)	0.2135
Arthralgia	5 (2.8)	1 (1.9)	0.7018	94 (16.9)	11 (6.3)	0.0005
Systemic rash	NA	NA	NA	2 (0.4)	0 (0)	0.4260
Pruritus	2 (1.1)	0 (0.0)	0.4353	16 (2.9)	1 (0.6)	0.0766
Facial paresthesia	2 (1.1)	0 (0.0)	0.4353	8 (1.4)	1 (0.6)	0.3620
Non facial paresthesia	3 (1.7)	0 (0.0)	0.3383	21 (3.8)	2 (1.1)	0.0806
Need for antipyretic or analgetic medication				189 (33.9)	32 (18.2)	<.0001

Table S3. Reactogenicity and immunogenicity following first and second vaccine by gender.

	After 1st Vaccination (N=233)			After 2nd Vaccination (N=733)		
	Female (N=179)	Male (N=54)	P	Female (N=557)	Male (N=176)	P
IgG - Anti RBD (AU), Geometric Mean (95%CI)	0.11 (0.08-0.16)	0.13 (0.07-0.22)	0.7	33.5 (33.85-37.2)	26.9 (24.1-30.1)	<.0001
Neutralizing antibodies, Geometric Mean (95%CI)	24.3 (18.5-31.9)	19.8 (8-49)	0.6	923.6 (734-1161)	362 (153-858)	0.04

* NA were done in 58 and 13 in females and males respectively after the first vaccine and in 94 and 24 females and males respectively after the second vaccine

Table S4. Reactogenicity and immunogenicity following first and second vaccine by age.

	After 1st Vaccination (N=233)			After 2nd Vaccination (N=733)		
	< 55 years old (N=176)	>55 years old (N=57)	P	< 55 years old (N=558)	>55 years old (N=175)	P
IgG - Anti RBD (AU), Geometric Mean (95%CI)	0.13 (0.09-0.18)	0.09 (0.06-0.13)	0.1226	35.62 (34.09-37.22)	26.62 (23.44-30.23)	<.0001
Neutralizing antibodies (NA), Geometric Mean (95%CI)*	25.4 (18.5-34.8)	18 (11-30)	0.2796	872.6 (655-1163)	588 (352-982)	0.1479

* NA were done in 54 and 17 HCW < 55 years old and > 55 years old respectively after the first vaccine and in 78 and 40 HCW < 55 years old and > 55 years old respectively after the second vaccine
